# Supplementary material for: Lipid-Encapsuled Grape Tannins Prevent Oxidative-Stress-Induced Neuronal Cell Death, Intracellular ROS Accumulation and Inflammation
Source: Antioxidants (Basel). 2022 Sep 28;11(10):1928. doi: 10.3390/antiox11101928 (PMC9598423; doi:10.3390/antiox11101928)
Supplement: Supplementary file 1 [file antioxidants-11-01928-s001.zip › antioxidants-1870070-supplementary.pdf]

antioxidants MDPI

Supplementary Material

Table S1. TS characterization by UV-HPLC Analysis.

Analysis	TS	TLS
	Concentration (mg/L)	Concentration (mg/L)
Gallic acid	3.2	2.9
Catechin (terminal)	71.3	64.8
Catechin Cysteine	66.9	60.9
Epicatechin	22.2	20.2
Epicatechin Cys.	24.7	22.5
Catechin gallate	46.8	42.6
Epicatechin gallate	3.1	2.8
Epitecatechin gallate cys.	8.7	7.9
Procyanidin B1(cis-trans)	6.1	5.5
Procyanidin B2 (cis-cis)	Under detection limit	Under detection limit
Gallolization (%G)	68.8	62.6
Mean Degree of Polymerization (mDP)	2.02	1.83

The identification of the compounds was carried out by means of a depolymerization of tannins in acidic methanol and in the presence of toluene-α-thiol or cysteamine hydrochloride (acid thiolysis) and subsequent HPLC-UV analysis using a wavelength of 280 nm.

Table S2. 18S housekeeping gene standardization.

Sample ID	CT	Delta CT	Mean	Fold-change
Control 1	11.43	-0.04	-0.14	1.10
	11.24	-0.23		
Control 2	12.00	0.53	0.25	0.84
	11.44	-0.03		
Control 3	11.50	0.03	-0.03	1.02
.	11.38	-0.09		
Control 4	11.11	-0.36	-0.22	1.16
	11.40	-0.07		
Control 5	12.00	0.53	0.16	0.90
	11.26	-0.21	**mean**	**1.00 ± 0.13**
**Geometric mean**	**11.47**			
H_2O_2 1	11.15	-0.32	-0.39	1.31
	11.02	-0.45		
H_2O_2 2	11.81	0.34	0.44	0.74
	12.01	0.54		
H_2O_2 3	12.00	0.53	0.28	0.82
	11.51	0.04		
H_2O_2 4	11.52	0.05	0.09	0.94
	11.60	0.13		
H_2O_2 5	11.04	-0.43	-0.24	1.18
	11.42	-0.05	**mean**	**1.00 ± 0.24 (n.s.)**
H_2O_2+TLS 1	11.90	0.43	0.07	0.95
	11.19	-0.28		
H_2O_2+TLS 2	11.73	0.26	-0.02	1.02
	11.17	-0.30		

H_2O_2+TLS 3	11.00	-0.47	-0.10	1.07
	11.75	0.28		
H_2O_2+TLS 4	11.37	-0.10	-0.25	1.19
	11.08	-0.39		
H_2O_2+TLS 5	11.59	0.12	0.42	0.75
	12.19	0.72	**mean**	**0.99 ± 0.16 (n.s.)**
H_2O_2+LS 1	11.08	-0.39	-0.15	1.11
	11.56	0.09		
H_2O_2+LS 2	12.08	0.61	0.52	0.70
	11.90	0.43		
H_2O_2+LS 3	11.08	-0.39	-0.15	1.11
	11.56	0.09	**mean**	**0.97 ± 0.24 (n.s.)**
H_2O_2+TS 1	11.75	0.28	0.09	0.94
	11.37	-0.10		
H_2O_2+TS 2	12.08	0.61	0.36	0.78
	11.59	0.12		
H_2O_2+TS 3	12.19	0.72	0.50	0.71
	11.75	0.28		
H_2O_2+TS 4	11.37	-0.10	-0.25	1.19
	11.08	-0.39		
H_2O_2+TS 5	11.59	0.12	-0.08	1.06
	11.19	-0.28	**mean**	**0.93 ± 0.20 (n.s.)**

Scheme 2. ^{CT} value of mean delta CT against the geometric mean of Control samples was used. qPCR reactions were performed in duplicate for each sample. One-way ANOVA and Bonferroni's post hoc test. n.s. indicates no significant

differences vs. Control.

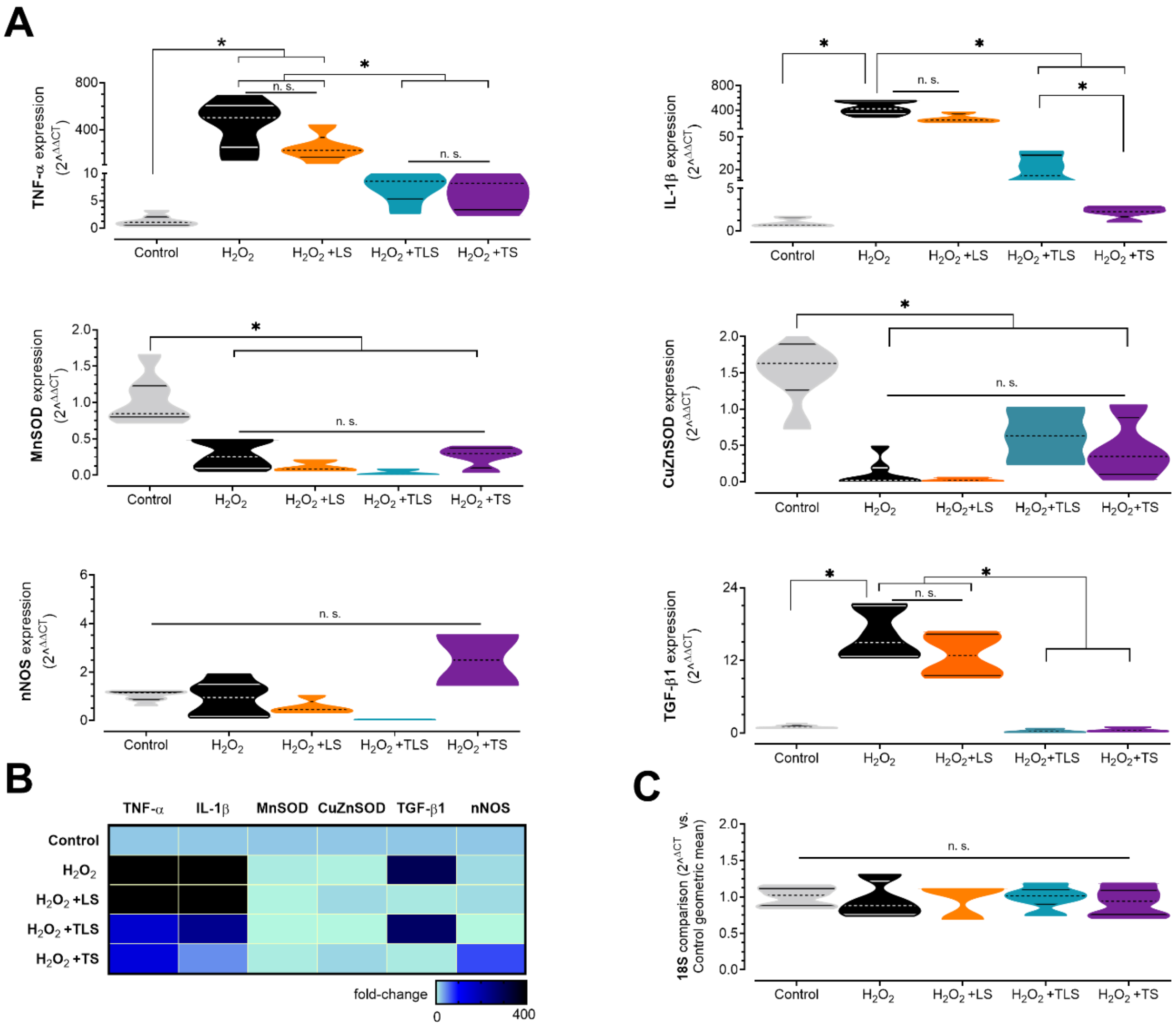

Figure S1. Effect of free tannins suspension and lipid-encapsulated grape tannins on neuroinflammatory biomarkers under oxidative stress conditions. A) Results of qPCR by the $2^{\Delta\Delta CT}$ method showing mRNA expression of proinflammatory biomarkers, antioxidant enzymes and anti-inflammatory biomarkers. B) Heat map showing the average mRNA expression (geometric mean) of gene targets evaluated in A). C) Graphical summary of 18S gene standardization after $2\text{-}^{\Delta CT}$ normalization of H_2O_2-treated cells against the geometric mean of Control CT values. n = 5 for Control, H_2O_2, H_2O_2+TLS and H_2O_2+TS; n = 3 for H_2O_2+LS, on which each n represents a different batch of CAD cells. One-way ANOVA and Bonferroni post hoc tests. *: $p<0.05$. Normal distribution was assessed by Shapiro-Wilk test (W values 0.8882; 0.9513; 0.8503; 0.9070; 0.8523; Control, H_2O_2, H_2O_2+TLS; H_2O_2+TS and H_2O_2+LS, respectively; $p>0.05$).
